# The HIV Mutation Browser: A Resource for Human Immunodeficiency Virus Mutagenesis and Polymorphism Data

**DOI:** 10.1371/journal.pcbi.1003951

**Published:** 2014-12-04

**Authors:** Norman E. Davey, Venkata P. Satagopam, Salvador Santiago-Mozos, Carlos Villacorta-Martin, Tanmay A. M. Bharat, Reinhard Schneider, John A. G. Briggs

**Affiliations:** 1 Structural and Computational Biology Unit, European Molecular Biology Laboratory (EMBL), Heidelberg, Germany; 2 Department of Physiology and Department of Biochemistry and Biophysics, University of California, San Francisco, San Francisco, California, United States of America; 3 Luxembourg Centre for Systems Biomedicine, Belval, Luxembourg; 4 Molecular Medicine Partnership Unit, EMBL/Universitätsklinikum Heidelberg, Heidelberg, Germany; National Cancer Institute at Frederick, Frederick, Maryland, United States of America

## Abstract

Huge research effort has been invested over many years to determine the phenotypes of natural or artificial mutations in HIV proteins—interpretation of mutation phenotypes is an invaluable source of new knowledge. The results of this research effort are recorded in the scientific literature, but it is difficult for virologists to rapidly find it. Manually locating data on phenotypic variation within the approximately 270,000 available HIV-related research articles, or the further 1,500 articles that are published each month is a daunting task. Accordingly, the HIV research community would benefit from a resource cataloguing the available HIV mutation literature. We have applied computational text-mining techniques to parse and map mutagenesis and polymorphism information from the HIV literature, have enriched the data with ancillary information and have developed a public, web-based interface through which it can be intuitively explored: the HIV mutation browser. The current release of the HIV mutation browser describes the phenotypes of 7,608 unique mutations at 2,520 sites in the HIV proteome, resulting from the analysis of 120,899 papers. The mutation information for each protein is organised in a residue-centric manner and each residue is linked to the relevant experimental literature. The importance of HIV as a global health burden advocates extensive effort to maximise the efficiency of HIV research. The HIV mutation browser provides a valuable new resource for the research community. The HIV mutation browser is available at: http://hivmut.org.

## Author Summary

Naturally occurring mutations within the HIV proteome are of therapeutic interest as they can affect the virulence of the virus or result in drug resistance. Furthermore, directed mutagenesis of specific residues is a common method to investigate the function and mechanism of the viral proteins. We have developed novel computational text-mining tools to analyse over 120,000 HIV research articles, identify data on mutations and work out which amino-acid in which protein has been mutated. We have organised these data and made them available in an online resource—The HIV mutation browser. The resource allows HIV researchers to efficiently access previously completed research related to their region of interest in the HIV proteome. The HIV Mutation Browser complements currently available manually curated HIV resources and is a valuable tool for HIV researchers.

## Introduction

Human immunodeficiency virus (HIV), the causative agent of acquired immunodeficiency syndrome (AIDS), infects millions of people worldwide and, to date, has been responsible for over 25 million deaths [1]. The clinical importance of the virus has prompted substantial funding of HIV/AIDS research across many diverse clinical, therapeutic (drug design, vaccine production) and basic research fields. This research has produced an extensive catalogue of HIV literature and consequently finding literature pertinent to a particular topic is a difficult task. Researchers are often interested in the phenotypic variation resulting from naturally occurring single nucleotide polymorphism or directed mutagenesis in the HIV genome. Traditionally, mutation data for a particular protein or region must be manually collected by trawling literature repositories such as PubMed using author names, protein/gene names, keywords or a mixture of all three. The scale of the HIV literature (over 270,000 articles) makes such an approach inadequate. Several valuable online resources have provided mutation data to researchers by manually curating polymorphism and mutagenesis data from HIV studies. These include the Stanford Drug Resistance database [2], which curates mutations related to drug resistance, the UniProt knowledgebase [3], which manually annotates articles describing mutagenesis of HIV proteins and the Los Alamos HIV Database, which annotates various sources of HIV data including epitope variants and escape mutations (http://www.hiv.lanl.gov/). However, these resources are limited in scope because manual curation cannot feasibly be carried out on all of the available literature.

The technology exists to quickly computationally scan, annotate and organise scientific literature and these techniques should be applied to facilitate the work of HIV researchers [4], [5]. Consequently, it is surprising that so few resources are available to access the available literature in an organised and structured way. This incongruity can partly be explained by the strict licensing agreements with scientific publishers that prohibit the bulk download and computational processing of scientific research literature. Fortunately, recent pressure from government and scientific bodies and the rise of open access publishing has softened the stance of publishers and many are now receptive to waiving these restrictions. Such advances will pave the way for many large-scale literature text-mining projects and will likely change the way we access scientific literature.

Here we have applied text-mining techniques to extract data on polymorphisms and mutations from the available HIV literature. We have organised this data in a protein and residue-centric way and have made it available through an online resource, the HIV mutation browser (http://hivmut.org). This publicly available resource will simplify the task of virologists attempting to identify the relevant literature for their research, thereby aiding experimental design and reducing replication of efforts.

## Results

**Figure 1:**
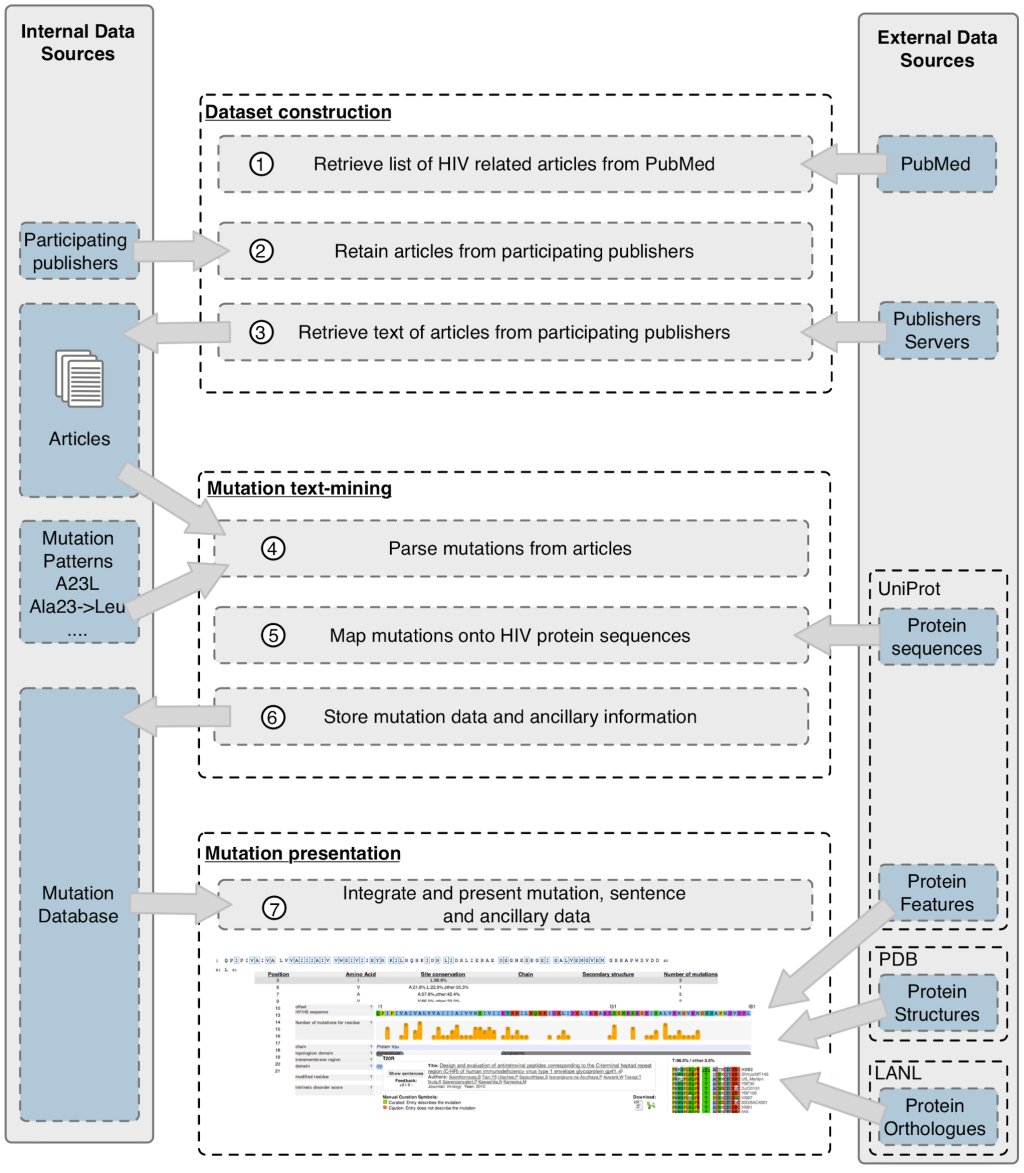
Schema describing article acquisition, mutation curation and data presentation for the HIV mutation browser. A list of HIV-related PubMed article identifiers (PMIDs) are retrieved from PubMed. The publishing journal of each article is compared against a list of participating publishers (i.e. publishers that have given permission for bulk PDF downloading, computational parsing of PDF and display of articles details). Permitted articles are retrieved from the publishers website as PDF files. Retrieved articles are computationally text-mined to parse patterns commonly used in the literature to denote mutations. Each mutation is then mapped onto the HIV proteome. Mutations are stored in a relational database and accessed through a web interface, the HIV mutation browser. The HIV mutation browser organises the data by protein and residue and integrates ancillary information relevant to the users. See methods section for more details.

Creation of the HIV mutation browser required a number of steps (Figure 1). First, we obtained permission from publishers, identified, and accessed the relevant literature. Second, we established and applied text-mining techniques to retrieve data on mutagenesis and polymorphism from the HIV literature. Third, we associated the mutation data to the appropriate residues within the HIV proteome. Finally, we developed a browser through which the data can be accessed in an intuitive and informative way.

### Data mining and mutation statistics

We identified ∼270,000 articles containing the search term “HIV” or “Human Immunodeficiency Virus” indexed in the PubMed database (from a total of ∼23 million publications). We retained 120,899 of these articles, published across 2,614 journals, representing approximately 45% of the total (see materials and methods, Figure 1). For the remaining ∼150,000 citations, permission for computational processing of articles was not obtained from the publisher. The 120,899 articles from participating publishers were text-mined for mutagenesis or polymorphism information, and the mutations were mapped to particular residues within the HIV proteome. This required the development of a method to retrieve the text of these articles, scan the articles for patterns that are widely used to describe directed mutations in mutagenesis experiments or polymorphisms, and to map these mutations to the correct position in the correct protein (see materials and methods). A total of 7,608 distinct mutations (a unique non-wildtype amino acid at a given residue in a given protein) were collected. As each mutation can be described in multiple articles and each article can describe multiple mutations, the 7,608 distinct mutations were defined by 43,264 unique references to 5,267 articles.

**Figure 2:**
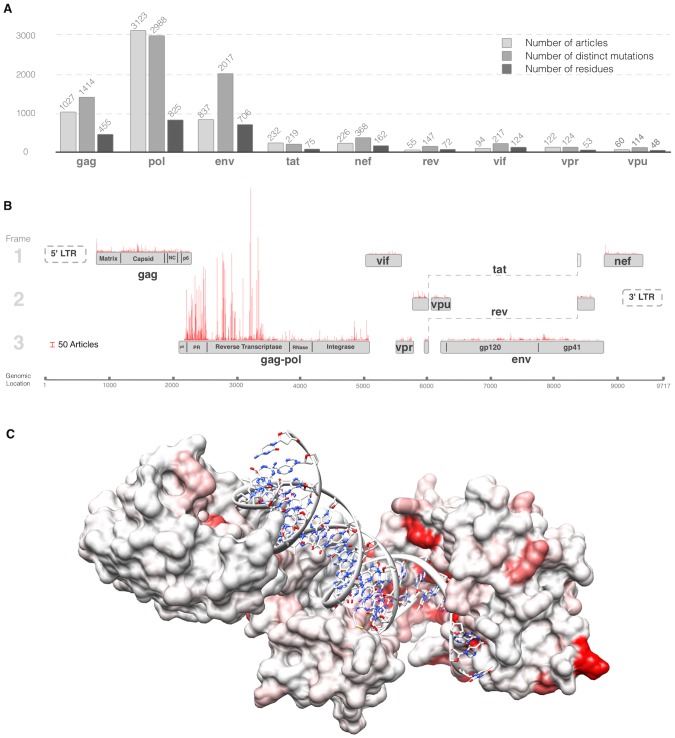
Overview of the distribution of mutation data across the HIV proteome. (A) Barplot of the counts of (i) the number of articles describing mutations, (ii) the number of distinct mutations and (iii) the number of residues with mutation data in the database for each protein in the HIV proteome. (B) Barplot of the counts of the number of curated articles in the database describing mutagenesis experiments or polymorphisms for each residue mapped onto the HIV proteome/genome. (C) Reverse transcriptase p66 subunit with residues coloured by number of articles referring to them. Most highly cited residues are in contact with the nucleotides or are known drug resistance mutations. White denotes no papers, full red denotes 50 or more papers, colouring is linearly scaled between 0 and 50.

The identified mutations shed light on the nature of the HIV research effort of the last decades. On the one hand it has been broad in scope: 2,520 of the 3,118 residues in the HIV proteome have one or more associated references to a mutation in the repository. On the other hand it has been narrow in focus: the coverage is far from uniform and certain regions such as the catalytic sites of the protease and reverse transcriptase, as well as host interaction interfaces, are much more highly studied (Figure 2).

### HIV Mutation Browser interface

The above analysis resulted in a database within which each reference to a mutagenesis experiment or polymorphism in a citation is indexed using three pieces of information: the protein in which the mutation is present, the position in the protein which has been mutated, and the non-wildtype amino acid to which the wildtype residue has been mutated. To make this data accessible to virologists in a simple, intuitive and informative manner, we designed the HIV Mutation Browser, as a web-interface that acts as a front end for the database. The browser presents the data in a hierarchically organised manner. The user selects a gene of interest, then a position of interest, and the citations relating to this position are presented to the user grouped by non-wildtype amino acid.

**Figure 3:**
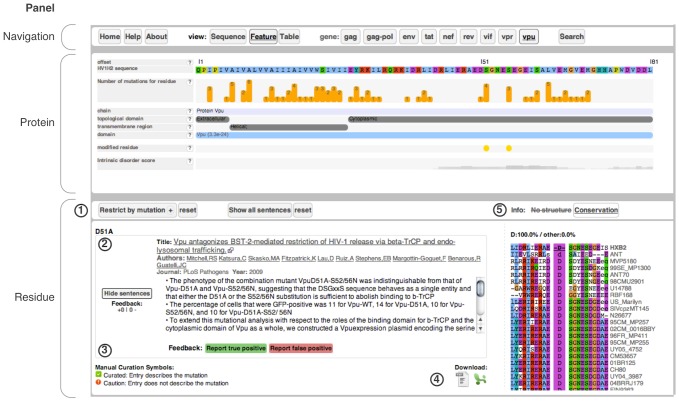
HIV Mutation Browser interface for Vpu residue 51 showing the navigation, protein and residue panels. (1) Options bar for the residue view section of the interface. (2) Mutation information. (3) User feedback buttons. (4) Mutation information download links. (5) Ancillary residue information panel.

The web interface is organised in three panels: the *navigation panel* at the top; the *protein panel* in the middle; and the *residue panel* at the bottom (Figure 3).

#### Navigation panel

The navigation panel (Figure 3) contains a set of buttons that allows users to navigate the site. The most important of these buttons allow users to access the mutation information by choosing the gene of interest (tagged *gene*) and one of three different options for visualising the data in the protein panel (tagged *view*). The search button gives users access to a tool to query the resource by full text, author and PubMed identifier. The left hand side of the panel contains links to access the home page, the help page (containing all the information necessary to understand the information displayed on the website) and the about pages (containing up-to-date statistics about the website and information about participating publishers).

#### Protein panel

The mutagenesis and polymorphism data can be visualised and accessed by the user in three ways corresponding to the Sequence, Feature and Table view. All views are accessible by clicking on the relevant button, tagged “view”, in the navigation bar (Figure 3). All views show the primary protein sequence of the selected HIV-1 gene - clicking on an amino acid of interest can access detailed mutagenesis data for that residue. The *Sequence* view displays the amino acid sequence of the protein of interest. Residues with mutation data are displayed in blue boxes. The *Feature* view displays the selected protein sequence annotated and enriched with known functional data, structural data and modular features (See Figure 3). The number of distinct mutations for each residue is displayed as a bar plot below the protein sequence. The *Table* view displays information on conservation, structure and chain, as well as the number of mutagenesis and polymorphism experiments available for each position in a tabulated format.

#### Residue panel

The residue panel displays the mutation information for the residue currently selected in the protein panel. The left side of the residue panel displays a list of citations that include mutation information for the residue. Each residue can have one or more distinct mutations and citations are grouped by the non-wild type amino acid to which the wild type amino acid has been mutated. Within these groupings, if more than one citation is present, the citations are presented in an order that is based first on the results of manual curation, then on user feedback (see below) and finally on number of views. Each distinct mutation in each article has a unique citation (i.e. the same article may appear multiple times for the same residue entry but with different non-wild type amino acids).

For each citation, the information presented includes details of the residue: its position in the reference proteome, the wild type amino acid and the non-wild type amino acid described in the article; as well as information on the article (title, author list, publishing journal, publication, year, direct link to the article at the publishers website). The ‘show sentences’ button reveals the sentences from the original article that describe the mutation and phenotype.

The right hand side of the residue panel contains supplementary contextual information. When available, the position of the residue is displayed on a solved structure of the protein domain, retrieved from RCSB Protein Data Bank (PDB) [6]. Hovering the cursor over the structure can rotate it to allow viewing from different directions. Alternatively, the user can choose to display a multiple sequence alignment of homologous reference HIV subtype protein sequences for the 10 flanking residues either side of the residue of interest.

#### User feedback and curation

The database has been populated by text-mining, and it is therefore unavoidable that the database contains incorrectly assigned citations (see discussion). We have therefore incorporated a user feedback system that allows users to flag the quality of an entry either positively or negatively. This feedback is presented for each entry and influences the order in which citations are presented to the user when multiple papers describing a given mutation are available. The feedback will also be used to guide manual curation by the HIV Mutation Browser team on a quarterly basis coinciding with each new releases of the HIV Mutation Browser. An emphasis will be put on the curation of entries that have received negative feedback and are likely to contain inaccuracies. Manually curated entries are indicated with a green tick (a good entry) or a red cross (a bad entry). This curation relates to the accuracy of the text-mining and does not reflect the quality of the paper.

### Download

The available mutagenesis and polymorphism data for a residue can be downloaded in both tab delimited text and Excel formats directly from the web interface.

## Discussion

HIV is an important therapeutic target and has been the subject of a major research effort as evidenced by the large catalogue of HIV experimental literature. Appropriate organisation and categorisation of the available HIV literature is necessary to allow efficient and intuitive access to relevant data. In this paper, we have presented the HIV Mutation Browser, a residue-centric resource of HIV mutagenesis and polymorphism literature designed for use by those carrying out basic and applied HIV research. The HIV Mutation Browser is one of the first resources to computationally text-mine mutagenesis and polymorphism data [7], [8], [9], and the first to apply such methods to the extensive corpus of HIV literature. As such the HIV Mutation Browser will complement the available manually annotated and curated HIV resources such as the Stanford Drug Resistance database [2], the UniProt knowledgebase [3] the Los Alamos HIV Database (http://www.hiv.lanl.gov/). In the coming years, we expect this method or similar methods to be applied to other viral or cellular systems.

The resource will continue to evolve in the following ways. Firstly, HIV literature is produced continuously at a rate of approximately 1,500 articles a month and consequently the HIV Mutation Browser resource will be updated on a quarterly basis. Secondly, while the resource does contain the majority of important HIV and general interest journals, it is still incomplete, as we did not receive permission from all publishers to text-mine their HIV related articles. Journals from additional publishers will be added when possible. Thirdly, not all mutations can be correctly identified and assigned by the text-mining methods. There are various reasons for this. Many mutations are annotated in an article using non-standard patterns that are not widely used to describe directed mutations in mutagenesis experiments or polymorphisms. For example, consider the following excerpt taken from an article by Mitchell et al., “The phenotype of the combination mutant VpuD51A-S52/56N was indistinguishable from that of Vpu-D51A and Vpu-S52/56N” [10]. The pattern “S52/56N” is a non-canonical construct for describing a mutagenesis experiment and currently will not be discovered by the text-mining method. Furthermore, the position of a mutation in a paper can be ambiguous and as a result mapping of the mutation information to the correct residue and protein can be a difficult task. For example, when multiple proteins are referenced and only a single mutation is discussed (more than one possible mapping can be possible), when unconventional numbering is used (particularly when describing mutations in Gag or Env as both are translated as polypeptide chains and subsequently cleaved) or when unusual strains with insertions and deletions are used (this shifts the numbering of residues). We will continue to improve the methods for text-mining and assignment. We request that members of the community utilise the feedback system for misannotated mutations in the resource and contact us about mutation data that should be in the resource yet is not present. This community input will improve the quality of the annotated data and will pinpoint parts of the text-mining method that require improvement.

In summary, the HIV Mutation Browser is a valuable addition to the currently available HIV resources that will allow researchers to quickly and intuitively access data on mutagenesis and phenotypic variation. We expect the database to aid the process of experimental design and be a key resource for the HIV community.

## Materials and Methods

### Construction of the HIV literature dataset

A list of HIV-related articles was programmatically retrieved from PubMed using the search terms “HIV” and “Human Immunodeficiency Virus”. A list of target journals was constructed based on the number of published HIV-related articles. The licensing agreements of the majority of scientific journals prohibit a licensee from (1) downloading articles in bulk and (2) computationally processing the text of an article. Permission to waive these aspects of the licensing agreement was requested and received from the majority of virology and general interest scientific journals. The text of all HIV-related articles from the participating journals was retrieved programmatically from the publisher's websites to create a *HIV literature dataset*. An up to date list of the participating journals and publishers is available on the HIV Mutation Browsers website.

### Mutation text-mining of HIV literature

**Figure 4:**
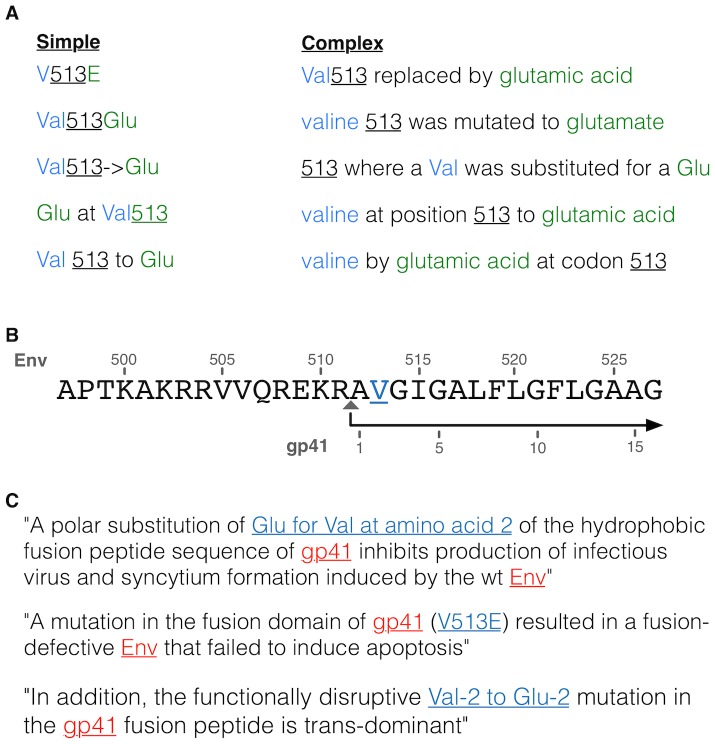
Example illustrations of issues associated with parsing and mapping mutation data. (A) Representative simple and complex examples of sentences recognised by the templates used to perform the mutation text-mining of articles (see Table S1 for complete list). Information of interest representing the wildtype residue (blue) and mutated residue (green) are coloured and position of the mutations are underlined. (B) Illustration of the distinct numbering schemes for different chains of the same protein. The shown peptide sequence is a short region (497–527) of the HIV Envelope glycoprotein gp160 (Env) overlapping the site cleaved by the host furin to produce the Surface protein gp120 and Transmembrane protein gp41 chains. The cleavage site is denoted by a grey triangle. The numbering above the sequence defines the position relative to the start of the gp160 protein and the numbering below the sequence defines the position relative to the start of the gp41 chain. (C) Examples of three sentences from the HIV literature where each article uses a different nomenclature or numbering scheme (blue) to describe the same mutation at the same site, Valine at residue 513 in the gp160 protein. Each article also refers to the protein by the chain name, gp41, rather than the name of the unprocessed protein, Envelope glycoprotein gp160 (Env), used for mapping in the HIV mutation resource. One example sentence refers to the gp41 chain while utilising the numbering for the unprocessed protein.

There is no globally applied nomenclature to define directed mutations in mutagenesis experiments or polymorphisms [5], [11], [12]. A set of templates that define phrases and shorthand widely used to describe directed mutations in mutagenesis experiments or polymorphisms was created based on the work of Caporaso *et al.* [5] (Figure 4A, see Table S1 for full list). Each article in the *HIV literature dataset* was converted to plain text and scanned using this set of templates. These templates consist of 3 pieces of information: the position in the protein which has been mutated, the amino acid present in that position in the wildtype sequence of the isolate, and the non-wildtype amino acid to which the residue has been mutated. For example, consider the sentence “*As reported previously, S52A and S56A mutations of Vpu had no effect on virus release*” [13]. S52A and S56A refer to the experimental mutagenesis of a serine to an alanine at position 52 and 56 in the Vpu protein.

### Mapping of mutations to the HIV proteome

The annotation of a mutation text-mined from papers in the *HIV literature dataset* requires three piece of information: the sequence of the isolate used in the study; the protein containing the mutation; and the position of the mutation within the protein. This information is sufficient to map a mutation to a reference HIV-1 proteome, but cannot always be directly extracted from the text-mined paper. The nomenclature for describing isolates, genes, proteins, chains and domains have not been standardised. Therefore, mapping dictionaries for HIV isolates and HIV proteins were constructed. The *isolate mapping dictionary* was constructed from isolate names and their synonyms retrieved from HIV data within the UniProt [3] and Allie [14] resources (Table S2). The *protein mapping dictionary* was constructed from synonyms for genes, proteins, cleavage products, chains and domain names from the HIV data, also retrieved from the UniProt [3] and Allie [14] resources (Table S3). The highly-studied HIV group M subtype B HXB2 isolate was selected as the reference proteome and all HIV genes, proteins, cleavage products, chains and domain names, and their synonyms, were mapped onto the 9 proteins of the isolate (Gag, Gag-Pol, Env, Tat, Nef, Rev, Vif, Vpr, Vpu). This mapping included normalised start positions to correct the inconsistent numbering schemes of cleavage products, chains and domain (Figure 4B). Both dictionaries were further manually curated to improve upon the computationally retrieved mapping.

Several different *experimental isolates* are commonly used in HIV experiments. Each paper in the *HIV literature dataset* was scanned using the contents of the *isolate mapping dictionary* to identify the *experimental isolate* used in the study (Table S2). If no isolate information was retrieved, the Human immunodeficiency virus type 1 group M subtype B HXB2 isolate was set as the *experimental isolate* for the paper. The numbering of a mutation in the HIV literature can refer to the numbering of a protein, chain, domain or cleavage product, consequently, for a defined mutation numbering a conclusive mapping may not possible. Inconclusively mapped mutations text-mined from the *HIV literature dataset* were mapped to the HIV proteome using a *co-occurrence based approach* (Figure 4C). The *co-occurrence based approach* utilised the Reflect tool for automated tagging of biological entities to scan mutation-containing sentences for protein identifying terms from the *protein mapping dictionary* [4]. Each mutation's position was normalised to the protein-numbering scheme of the full-length protein based on the co-occurring protein identifying terms. If the mutation wildtype amino acid matched the amino acid at the normalised mutation position in the *experimental isolate*, the mutation was retained as a mapped mutation. In the cases where no information relating to the mutated protein was available, all HIV HXB2 proteins were scanned at their full-length protein, chain and domain levels. In the case of chain and domain, a displacement factor was applied to adjust the mutation's position and map the mutation to all possible positions in the proteome. A mutation mapping score (see below) was calculated for each putative mutation mapping and the top scoring mapping was retained as the mapped position of the mutation. In the cases where no matches to the experimental isolate proteome were found, the search was expanded to other commonly studied HIV isolates (Table S2).

### Scoring mutation matches

For each mutation mapped using the above approach, a mutation mapping score, S, is calculated. The score is the function of three parameters: the probability of a match by chance; the number of mapped mutations in the paper; and the displacement from the reference protein position numbering scheme. The score ranges from 0 to 1, with values closer to 1 representing high confidence mapping of a mutation. The top-scoring mapping was retained as the mapped position of the mutation.

The score, S, is calculated as:
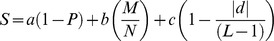
where *M* is the number of mapped mutations in the paper, *N* is the total number of mutations mentioned in the paper, *d* is the distance between the defined mutation position and the mapped position, *L* is the sequence length of the protein, the values of *a*, *b*, and *c* are constants to weight the contribution of each parameter in the equation (*a* = 0.7, *b* = 0.15 and *c* = 0.15) and *P* is the probability that the mapped mutation would map to the protein by chance and is calculated as:
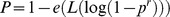
where *p* is 0.05, the probability of matching an amino acid by chance given a 20 amino acid alphabet and assuming an equal frequency for each amino acid in the HIV proteome, and *r* is the number of mutations that have been mapped unambiguously to the protein.

### Sources of ancillary data

The HIV Mutation Browser interface integrates information from several resources to increase the ease of interpretation of the available HIV mutation and mutagenesis data. Conservation information is displayed using multiple sequence alignments (aligned using the MAAFT algorithm [15]) retrieved from the HIV Subtype Reference Protein sequences from the Los Alamos National Laboratory (http://www.hiv.lanl.gov/). Structural information is displayed using structures of HIV proteins retrieved from the RCSB Protein Data Bank (PDB) [6]. Intrinsic disorder predictions for the proteins are calculated using the IUPred algorithm [16]. Enzymatic active sites, sites of post-translational moiety addition, sites of proteolytic cleavage and other sites of functional importance are retrieved from the UniProt resource [3]. Short linear motif interaction interfaces are retrieved from the ELM databases [17]. An up to date list of the ancillary information used and displayed is available on the HIV Mutation Browsers website.

## Supporting Information

Table S1 **Regular expressions for mutation identification and harmonization.** A list of templates/regular expressions, based on the work of Caporaso et al. [5], used to search the publications for sentences describing mutagenesis experiments or polymorphisms.
(XLSX)

Table S2 **The nomenclature for HIV isolates.** The dictionary of HIV strains/isolates and their corresponding synonyms, used to identify the HIV isolates mentioned in the publications.
(XLSX)

Table S3 **The nomenclature for HIV genes, proteins, chains and domains.** The dictionary of HIV genes, proteins, cleavage products, chains, domains and their synonyms.
(XLSX)
